# Trace Explosives Detection by Photoluminescence

**DOI:** 10.1100/tsw.2004.7

**Published:** 2004-02-26

**Authors:** E. Roland Menzel, Kimberly K. Bouldin, Russell H. Murdock

**Affiliations:** Center for Forensic Studies, Texas Tech University, Lubbock, TX 79409-1051, USA

**Keywords:** explosives, color tests, lasers, photoluminescence, fluorescence, ETK^Plus^, lanthanides, europium, terbium, nanoparticles, time-resolved imaging

## Abstract

Some field tests in counter-terrorism efforts to detect explosive traces employ chemistries that yield colored products. We have examined a test kit of this kind, ETK, based on widely used chemistries and employed extensively by the Israel Police. Our investigation focuses on the prospect of gaining sensitivity by replacing the normal colorimetric modality with photoluminescence detection, which, to our knowledge, has not been explored to date. We find two or more orders of magnitude sensitivity gains for all explosives studied, using field-worthy photoluminescence techniques. We have also investigated a general lanthanide-based photoluminescence approach which shows promise and the ability to photoluminescence-detect trace explosives in the presence of intense background color and/or background fluorescence by time-resolved imaging.

## INTRODUCTION

Counter-terrorism efforts include the detection of explosives, either in bulk or in trace amounts. The former might involve the screening of luggage at airports and would employ expensive non-portable instrumentation, perhaps 3-dimensional x-ray imaging, detection based on neutron activation, etc. Analytical methodologies for trace explosives detection may involve gas chromatographs, infrared instruments, or ion mobility spectrometers. True field-detection of trace explosives employs bomb-sniffing dogs or utilizes color spot test kits, which are simple, rapid and relatively cheap, and thus could be envisioned to be deployed as widely as, say, the breathalyzer or radar gun found in many police vehicles. Indeed, the ETK$^{Plus}$ (Explosives Detection Kit)[1,2], manufactured by the Israel Institute for Biological Research, Ness-Ziona, Israel and marketed in the U.S. (Homeland Security Products, LLC, 307 East Shore Rd., Great Neck, NY 11023, Tel: 516-466-5210), is extensively used by the Israel Police and some other law enforcement agencies to field-detect traces of explosives on the hands of suspects for instance. ETK has recently been used to assess the detectability of explosive traces after immersion in water[3]. This pertains to airplane explosion followed by crash in the ocean, for instance. Similarly, field screening of trace amounts of explosives is of interest in assessment of environmental contamination; here, again, colorimetric tests based on chemistries similar to ETK are employed[4]. The low cost, ease of use, and speed of color tests come, however, at the price of low sensitivity compared to the more sophisticated laboratory analyses such as gas chromatography–mass spectrometry. It therefore becomes compelling to investigate means of increasing the sensitivity of colorimetric field detection of trace explosives without serious detriment to cost, simplicity, and speed; this is the topic of our study. It involves the exploitation of the photoluminescence phenomenon to gain sensitivity over colorimetry[5,6,7]. Photoluminescence here refers to luminescence, namely emission of light, either as fluorescence or phosphorescence, produced by prior absorption of light of a color different—of shorter wavelength—from the emitted color.

To set the stage for field detection of trace explosives by photoluminescence techniques, we briefly digress to consider an analogous problem, namely, the detection of latent fingerprints. For over 40 years, the detection of these prints on items such as paper has relied on as the workhorse the reagent ninhydrin, which targets amino acids of the fingerprint residue to form a purple-blue product, Ruhemann's Purple (RP), so named after English chemist Siegfried Ruhemann, who first synthesized ninhydrin in 1910 and recognized its reaction with amino acids. The ninhydrin method is colorimetric. The nonfluorescent RP can be converted to a fluorescent form upon a simple subsequent treatment with zinc chloride[8] to yield a massive gain in sensitivity. Indeed, photoluminescence-based fingerprint detection in a variety of modalities is employed worldwide today owing to its superior sensitivity over the corresponding colorimetric detection procedures, be they dusting, staining, or chemical[9]. However, the detection via photoluminescence implies the need for a light source that excites the photoluminescence. In the fingerprint context, high-power lasers were initially employed[10] to achieve maximum sensitivity because photoluminescence intensity is proportional to illumination intensity. Filtered lamps—cheaper, easy to use, and portable—were examined from the outset as well[10]. The cost and ease of use gains come at the price of sensitivity, however, because the useful powers delivered by such lamps are much lower (typically about 500 mW) than those delivered by high-power lasers (5 W or more). Nonetheless, filtered lamps, often referred to by forensic practitioners as alternate light sources or forensic light sources, have since become commercial and are now in wide use. Any photoluminescence approach to trace explosives detection in the field must consider light source requirements (intensity, price, portability, simplicity) as well.

## LIGHT SOURCES

The light source must deliver the appropriate color to be absorbed by the material that is to photoluminesce. The so-called alternate light sources used for fingerprint work and other evidence examinations (documents, fibers, body fluids) typically come with filter wheels (equipped with a series of band-pass filters) to extract the appropriate color of light. They are portable, in the sense that they operate on ordinary household current or via a converter that changes an automobile battery output to 110 volts AC. However, they are bulky and expensive, and thus are perhaps not ideal in a field scenario. There are a number of portable (bulky) high power lasers on the market, but they are prohibitively expensive for application only in the kind of widespread field scenario of trace explosives detection with which we are presently concerned. However, if the explosives application is just one of the uses of such portable lasers, then there is no obstacle. Diode lasers, even light-emitting diodes, which operate in a wide range of colors, come to mind as potentially applicable light sources, but currently are slightly lacking in power. Frequency-doubled CW (as opposed to pulsed) Nd:YAG (or Nd:$YVO_4$) lasers, which deliver green light at 532 nm and are battery operated, flashlight-sized, and operated with flashlight ease, are currently on the market, with powers adequate for our purposes. These green flashlight-like lasers currently find mostly military applications. They are especially interesting in photoluminescence detection of explosives. Finally, there are inexpensive hand-held ultraviolet lamps. They can operate either in the near UV (wavelength >300 nm) or the deep UV (wavelength <300 nm) spectral ranges. The chemistries currently used in colorimetric detection of explosives mostly yield blue, pink-red, red, or red-violet test spots. If these colored products were photoluminescent, they would call for orange-red excitation in the case of blue products and blue-green excitation in the case of the reddish products. Explosives detection chemistries that respond to UV excitation also are possible. Here, we focus on photoluminescence methods that respond to green or UV excitation. This, as we shall see, is not a serious restriction in that most types of explosive can be handled with these excitations.

## COLORIMETRIC EXPLOSIVES DETECTION CHEMISTRIES

Many of the colorimetric explosives detection chemistries in current use are actually quite old, dating back to the 19$^{th}$ century (e.g., Janowsky, 1886, 1891; Griess, 1864, 1879; Franchimont, 1897; Meisenheimer, 1902). They have become the stuff of textbooks[11,12,13]. Alkali-based methods are reported to yield red or reddish products with Type I explosives, such as 2,4- dinitrotoluene (DNT), trinitrotoluene (TNT), 2,4,6-trinitrophenylmethylnitramine (tetryl), etc. Alkaline hydrolysis producing nitrite ions detected by the Griess reaction probes Type II explosives, such as nitrocellulose (as in smokeless powder), nitroglycerin (NG), cyclotrimethylenetrinitramine (RDX), and many other military-type explosives, again yielding reddish products. Similarly, Type III explosives containing inorganic nitrates, as in black powder or ANFO (a mixture of ammonium nitrate and fuel oil), yield red products. Since these chemistries are used on trace amounts of explosive, it stands to reason that the colored products have high molar extinction coefficients (i.e., are intensely colored), and thus should fluoresce, barring structural problems, such as poor $\pi$ orbital overlap or the presence of an element of high atomic number, leading to fluorescence quenching by intersystem crossing[9]. The higher the molar extinction coefficient, the shorter the radiative fluorescence lifetime, hence the lower the prospect that nonradiative processes can compete with fluorescence[14]. Nonetheless, to the best of our knowledge, the luminescence properties of the previously mentioned colored spot tests have not been exploited to date.

## ETK METHOD

The kinds of chemistries discussed above are incorporated in the commercially available aforementioned ETK$^{Plus}$ kit. The commercial availability of any such test kit is essential. The test kit probes a wide range of explosives and thus serves as a good vehicle to compare colorimetric and photoluminescence detection of explosives in a general sense.

### Procedure and Results

Samples of RDX were obtained from the Lubbock County Bomb Squad. Nitroglycerin tablets, as prescribed for certain cardiac diseases, served as the source of NG. Black powder (inorganic nitrate as the active ingredient) and smokeless powder (single-base, i.e., with nitrocellulose as the active ingredient) were purchased from local gun shops. The explosive material was extracted

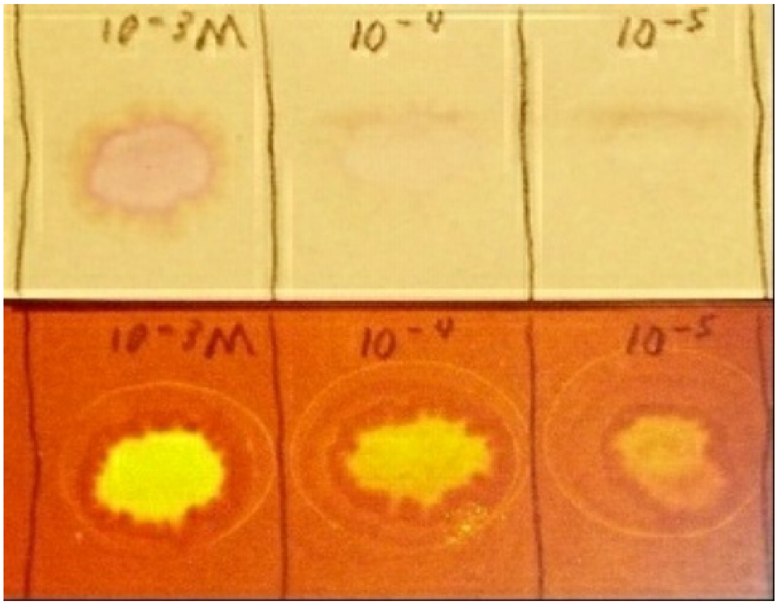

**FIGURE 1.** Sensitivity comparison for RDX by colorimetry (top) and photoluminescence (bottom) using ETK$^{Plus}$. See text. Chromatography paper strip width = 3.8 cm.

with acetone (the usual solvent for this work) and spotted on 1Chr Whatman chromatography paper strips (devoid of fluorescent brighteners; filter paper can be substituted) in successive dilutions by factors of 10. The spots were then treated with ETK as prescribed by the manufacturer and the visual colorimetric detection was compared with photoluminescence visual detection (observation in a darkened room through an orange laser safety filter), utlizing blue-green Ar-laser light of 40 mW/cm$^2$ intensity. The photoluminescence detectability in all cases was two or more orders of dilution better than the colorimetric detection limit. Fig. 1 shows a photographic comparison (worst case observed) for the case of RDX. The top part of the photograph shows the colorimetric dilution result and the bottom the corresponding result in the photoluminescence mode. The top part of the photo was taken in room light with a digital camera and the bottom part was taken in absence of ambient light, under the Ar-laser excitation mentioned above, with the camera equipped with an orange 550-nm long-wavelength-pass filter that blocks scattered laser light but transmits the luminescence of interest. The photographs were converted to black and white, for ease of copying or printing. It should be noted that visualization (or photography) has to be made within a few minutes of the ETK spotting because in both the colorimetric and photoluminescence modalities, control samples (absence of any explosive) develop color and luminescence (due to the ETK reagents themselves) in time spans of about 10 min. This has been reported by others as well[3]. Note the bottom right spot of Fig. 1. It represents the fluorescence detection limit. In this spot the ETK spotting extended at the lower right beyond the explosive spot. Thus, this lower right portion serves as a control. The control locale luminescence is less intense than the explosive locale luminescence and also differs in color, namely, more orange and less yellow, from the explosive luminescence. The results obtained with the Ar-laser illumination (blue-green, all lines) were comparable to those obtained with frequency-doubled Nd:YAG (CW) laser illumination at the same intensity. This is of interest in terms of the commercially available flashlight-like green laser (100 mW/cm$^2$) mentioned earlier (e.g., Model 532 GBD, B. E. Meyers & Co., Inc., 14540 NE 91$^{st}$ St., Redmond, WA 98052, www.BEMEYERS.com).

We did acquire from the Lubbock County Bomb Squad a sample of military-type TNT "dynamite" and obtained with it results comparable to the other investigated explosives. We are told, however, that this kind of TNT dynamite contains as the active ingredient not only TNT, but other explosive as well. Because we were not able to procure a pure sample, we cannot interpret our results as definitive for TNT. We did, however, examine 2,4-DNT (Aldrich). To our surprise, spotting with tube 1 as prescribed in the

ETK procedure immediately produced a green, non-fluorescent product. This product eventually changed color to grey and eventually became reddish, and during this course remained nonfluorescent. The times involved in these subsequent color changes are too long to be of practical value. However, when the initial green product was spotted with tube 2 of ETK (this is not the normal procedure for Type I explosive) the green product quickly became orange and highly fluorescent under Ar-laser excitation.

To assess optimal excitation and optical filtering for fluorescence visualization, we carried out excitation and fluorescence spectral measurements, focusing on Type II explosive, which we consider to be the most important type. All explosives of this type should spectroscopically behave the same way, in that the common chemical feature is the production of nitrite ions. We utilized smokeless powder and spotting on chromatography or filter paper. The excitation source was a xenon arc lamp followed by a 0.25 m monochromator. The sample fluorescence was processed by a 0.3-m monochromator equipped with a photomultiplier tube of extended S-20 spectral response and photon counting instrumentation. At high spotting concentrations, the observed fluorescence, broad and featureless, peaked at about 605 nm with full width at half maximum (FWHM) of about 70 nm. The corresponding excitation spectrum, also broad and featureless, peaked at about 550 nm with FWHM of about 140 nm. At reduced concentrations, the fluorescence peak blue-shifted (a common occurrence to about 575 nm with the same FWHM.

## LANTHANIDE SPECTROSCOPIC AND CHEMICAL PROPERTIES

The lanthanide elements typically exist in compounds in the trivalent state. Some of them, most notoriously europium and terbium, $Eu^{3+}$ and $Tb^{3+}$, luminesce with high quantum efficiency. The most intense luminescence of the europium ion occurs at about 615 nm (red), arising from the transition from the upper $^5D_0$ to the lower $^7F_2$ state. For terbium, the corresponding states are $^5D_4$ and $^7F_5$, respectively, with green luminescence at 545 nm[9,15]. Although the luminescence efficiencies can be high, the luminescence intensities are generally quite low because the lanthanide molar extinction coefficients (proportional to the ion’s ability to absorb light) are very low indeed; this is due to the fact that the transitions from the ground state ($^7F_0$ for the europium and $^7F_6$ for the terbium ion) are both parity- and spin-forbidden[16]. Moreover, many lanthanide compounds include waters of hydration that quench the lanthanide photoluminescence via coupling of the O-H vibrational overtones to the lanthanide electronic states in much the same way as molecular vibrations couple to electronic states to quench molecular fluorescence via the well-known internal conversion and intersystem crossing mechanisms[9,16]. The severity of the quenching is proportional to the number of waters of hydration in the compound[15]. As early as 1942, it was discovered[17] that very intensely luminescent lanthanide compounds can be prepared by binding to the lanthanide ion organic ligands that (a) occupy all binding sites of the lanthanide ion, thereby excluding waters of hydration, and (b) absorb well and then transfer this excitation energy to the lanthanide ion (much more effectively than direct absorption by the lanthanide ion itself) via the Forster energy transfer process[18]. Such lanthanide complexes have become widely used in various bioscience fields and even in latent fingerprint detection[9]. Typically, the excitation of the lanthanide (either europium or terbium) complex is in the ultraviolet (to the ligand and from there to higher lanthanide states via the Forster process, followed by radiationless decay within the lanthanide ion to the lower-lying emitting lanthanide excited state, followed by the lanthanide luminescence). Action by the ligand corresponding to the direct energy transfer of the excitation to the emitting state (579 nm for the europium and 488 nm for the terbium) is not effective because this (lower) excited state does not couple well to ligand states (on parity grounds), and hence is not readily amenable to the ligand-lanthanide energy transfer. The absorbing ligand that transfers the excitation energy to the lanthanide ion is often referred to as a sensitizing ligand.

In colorimetric detection schemes, the pertinent properties of light (via absorption/reflection) are color and intensity. In photoluminescence schemes, the pertinent properties of the emitted light are color and intensity as well, but there is now a third property that can be exploited, namely, luminescence lifetime, the time of decay of the luminescence once the excitation (illumination) is shut off. Lifetimes of typical molecular fluorescences are on the order of a nanosecond. Phosphorescences have much longer lifetimes. The lanthanide luminescences rightly can be classified as phosphorescences because of their long lifetimes—of millisecond order—and because the two states involved in the emission process have different spin multiplicities[9,16]. The long lanthanide luminescence lifetimes permit detection in the presence of strong background fluorescence by time-resolved techniques[9], and thus are especially interesting, not only in the forensic science context but in many other fields as well.

Lanthanides have ninefold full coordination. This is easily seen by noting that common lanthanide salts are of the form $LX_3 \cdot 6H_2O$, where L is the trivalent lanthanide cation ($Eu^{3+}$ or $Tb^{3+}$ for our purposes) and X is a monovalent anion (chloride or nitrate most often), with 6 water molecules completing the coordination. The bonding of the water to the lanthanide ion through the oxygen end of the water is neither ionic nor of the typical covalent bonding variety of organic molecules. It has some electrostatic characteristics, namely, the attraction between a charged object and an electric dipole, reminiscent of hydrogen bonding, and some covalent characteristics as well. Many of the organic ligands useful for making highly luminescent lanthanide complexes are bidentate (occupying two coordination sites). Thus, only four such ligands can bind to the lanthanide, leaving one free coordination site which may remain unoccupied if the ligands are large, so that steric hindrance precludes access by water or another kind of ligand to this last site, or the site is occupied by a monodentate ligand. The one of most concern here is water, a notorious lanthanide luminescence quencher. One water of hydration may still be tolerable, but a larger number of waters of hydration very seriously degrades lanthanide luminescence, the quenching being proportional to the number of hydration waters[15]. When lanthanides form coordination complexes, they show a preference for binding to oxygen, as in water, for instance[15]. This preference may increase further when the oxygen acts as if it were a negatively charged entity, as it does in most explosives via the ubiquitous $NO_2$ functionality, as seen in Fig. 2 (the structure of RDX). The proclivity of lanthanides for water is such that a number of lanthanide complexes that are highly luminescent are quenched once placed in the presence of water because the water displaces the sensitizing ligands.

## LANTHANIDE METHOD

We use in our study lanthanide complexes with sensitizing bidentate ligands attached in the following modality. When the lanthanide complex comes in contact with a molecule of $NO_2$ – containing explosive it either binds to one (or both) oxygen(s) of the $NO_2$ of the explosive, which acts as a monodentate (or bidentate) ligand. In the later case, there is a displacement of a previously present ligand by an $NO_2$ moiety of the explosive. This completes the coordination, creating a hydrophobic environment around the lanthanide ion impervious to attack by water. We cannot tell from the results presented below which of these alternatives pertains, but this is not critical in that in either case there is a bona fide binding of the lanthanide complex to the explosive and an exclusion of water. If the sensitizing ligand is properly chosen, then exposure to water of the complex not bound to the explosive results in ligand displacement and consequent lanthanide photoluminescence quenching. Thus, our test procedure consists of spotting acetone-extracted

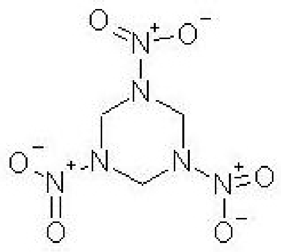

**FIGURE 2.** Structure of RDX.

explosive on chromatography paper, as usual, then spotting the paper with, or immersing it in, a solution (methanol) of the lanthanide complex, and finally subjecting the paper to a water rinse. At the explosive spot site, intense lanthanide luminescence is seen. Elsewhere it is quenched via the water rinse. The water rinse does not wash the unreacted complex away, but rather displaces ligands from it, so that waters of hydration replace them, quenching the luminescence. This is evident when one rinses with methanol instead, the methanol being a better solvent of the complex than water, for that matter. After the methanol rinse, in both the reacted and unreacted regions of the paper, the lanthanide luminescence persists because there is no water to quench luminescence. This rinsing distinction is important because one might otherwise consider that the lanthanide complex does not bind to explosive but simply is not as easily washed away from the somewhat nonpolar explosive spot site environment (at very high explosive concentration, a water drop placed at the explosive spot site has a tendency to bead) as from elsewhere on the paper. The rinsing situation is the same on bare chromatography paper even for spotting from methanol solution lanthanide chloride hexahydrate (which luminesces, but not strongly) in absence of any sensitizing ligand. The lanthanide salt and lanthanide complexes with sensitizing ligands have an affinity for paper and are thus immobilized and not rinsed off the paper. Thus, in the case of the lanthanide complex with sensitizing ligands, the luminescence quenching can only come about by sensitizing ligand displacement by water, and this displacement is inhibited only when free coordination sites of the lanthanide are taken up by the explosive. Fig. 3 shows how the two kinds of sensitizing ligand investigated by us bind to lanthanides ($L^{3+}$ in the figure). They have previously shown their mettle in the fingerprint context[9,19]. They are thenoyiltrifluoroacetone (TTFA) and ortho-phenanthroline (OP). To our knowledge, lanthanide compounds have not to date been investigated for the detection of explosives.

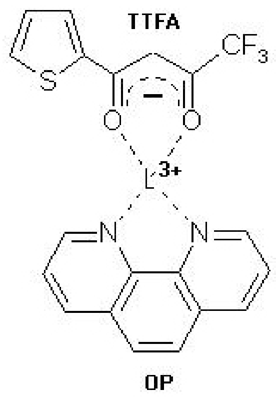

**FIGURE 3.** Binding of OP and TTFA to lanthanides.

## Procedure and Results

$Eu(TTFA)_4$, $Eu(OP)_4$ and the corresponding terbium complexes were prepared quite simply by mixing the commercially available (Aldrich) lanthanide chloride hexahydrate salt and TTFA or OP in methanol. A fivefold excess of ligand was used over the 1:4 lanthanide:ligand stoichiometric proportion. In our study, the resulting concentration of the complex was about $3 \times 10^{-4}$ M. The chromatography paper spotted with the explosive was then immersed for a second or two in the methanol solution of the lanthanide complex, or was spotted with the lanthanide complex solution. When the paper was left to dry for a minute or two, the result shown in Fig. 4a emerged for the europium complex with OP and smokeless powder, under the deep UV excitation pertinent to OP and visual inspection without any kind of filter. We observed around the intensely luminescent (red) region where the explosive is located a light blue-luminescent halo, which arises either from displacement of residual water from the explosive spot region via the methanol solvent or from migration of the excess OP used in the formulation of the lanthanide complex. In the halo region, that water, in turn, would displace ligands from unreacted complex to quench luminescence and/or the excess OP would dominate the luminescence of the halo region. Thus, in the halo region the observed luminescence was that of free OP (light blue). Farther out from the halo region, intense (red) luminescence from unreacted lanthanide complex was seen, together with the light blue luminescence of free OP due to the excess of OP in the formulation of the lanthanide complex. The overall effect in this region was the observation of a pinkish red luminescence. When the sample was inspected through a band-pass filter tuned to the red europium emission (wedge filter), no luminescence was seen from the halo region and the explosive spot region and the region of unreacted complex showed the same intensity, at least to the level of what could visually be discerned. When the sample shown in Fig. 4a was subsequently rinsed in running tap water for a few seconds (or was simply immersed briefly in water), the resulting Fig. 4b was obtained, with no luminescence seen from unreacted complex. It is not necessary to let the paper dry before observation, nor is it necessary to let the paper dry for any length of time after the prior methanol spotting or immersion. Thus, the procedure is quite rapid. The results of Figs. 4a and 4b were again recorded by digital photography, the camera being equipped with a pale yellow 470-nm long-wavelength-pass filter (used to block unwanted reflected residual violet and UV). The illumination employed a hand-held UV lamp (Model UVGL-58, Mineralite® Lamp, UVP,

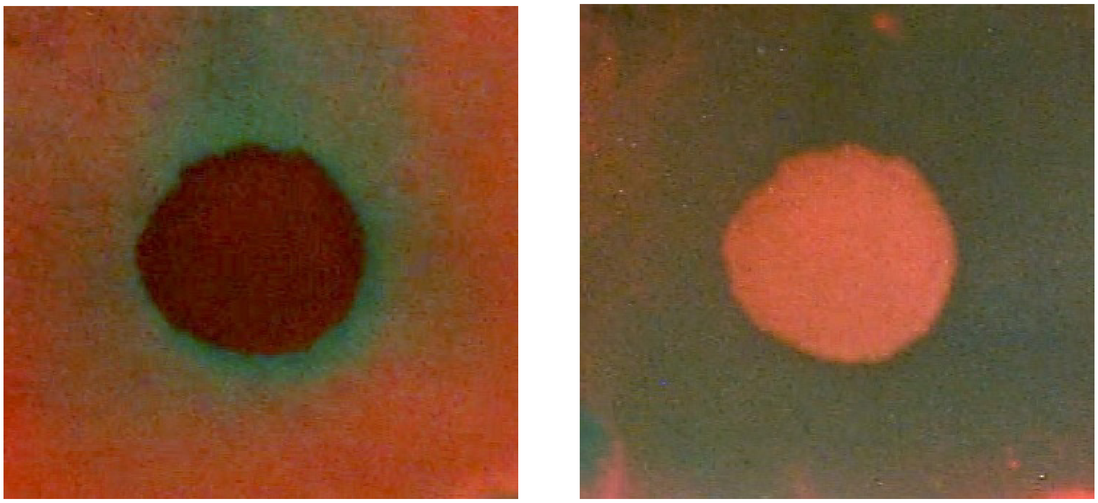

(a) (b)

**FIGURE 4.** Smokeless powder spot on chromatography paper treated with europium-OP before water rinse (a) and after water rinse (b), as detected by photoluminescence. See text.

Upland, CA) operating in the deep UV, and were then converted to black and white, with blue suppression, because the luminescence of free OP is not of interest. Results obtained with RDX were similar to the above smokeless powder ones. Results were also comparable when the europium complex was replaced by the corresponding green-emitting terbium complex, for both smokeless powder and RDX. Results were similar, but not nearly as good, with the corresponding TTFA complexes (under near UV), because the TTFA ligand acts somewhat as a negatively charged moiety (OP acts neutral), which tends to inhibit the binding (electrostatic repulsion) to the explosive (whose binding oxygen also displays negative charge characteristics) and because the TTFA ligand is not as easily displaced by water as the OP ligand, binding more strongly to the lanthanide cation (electrostatic attraction). Under the near UV excitation appropriate to the TTFA ligand and study of smokeless powder, the powder spots themselves (acetone extraction) on the chromatography paper exhibited a green fluorescence of the same color (on visual inspection) as the Tb emission, though dim in comparison. In the sensitivity study below we focused on the Eu complex with OP, and smokeless powder and RDX, spotted as before in successive dilutions by factors of 10. The room light ETK$^{Plus}$ colorimetric smokeless powder result is shown at the top of Fig. 5 and the luminescence result (under deep UV and with the above filter) at the bottom. The left-most spots at the top and bottom of the figure do not correspond to color or luminescence. They are controls (changes of paper reflectivity characteristics due to solvent spotting). Somewhat disappointingly, the luminescence sensitivity was found to be about an order of dilution less than the ETK colorimetric sensitivity, as with RDX also. We note, though, that the illumination intensity was very low (about 1 mW/cm$^2$); that this is only an initial investigation that surely can be improved upon, just as this took place in the fingerprint context[9], in terms of chemical protocol (solvent, pH, substrate choice, etc.) and choice of sensitizing ligand; and that one of its unique purposes is *in situ* application in the presence of interfering background color (masking colorimetric detection) or background fluorescence (masking detection in a normal photoluminescence modality).

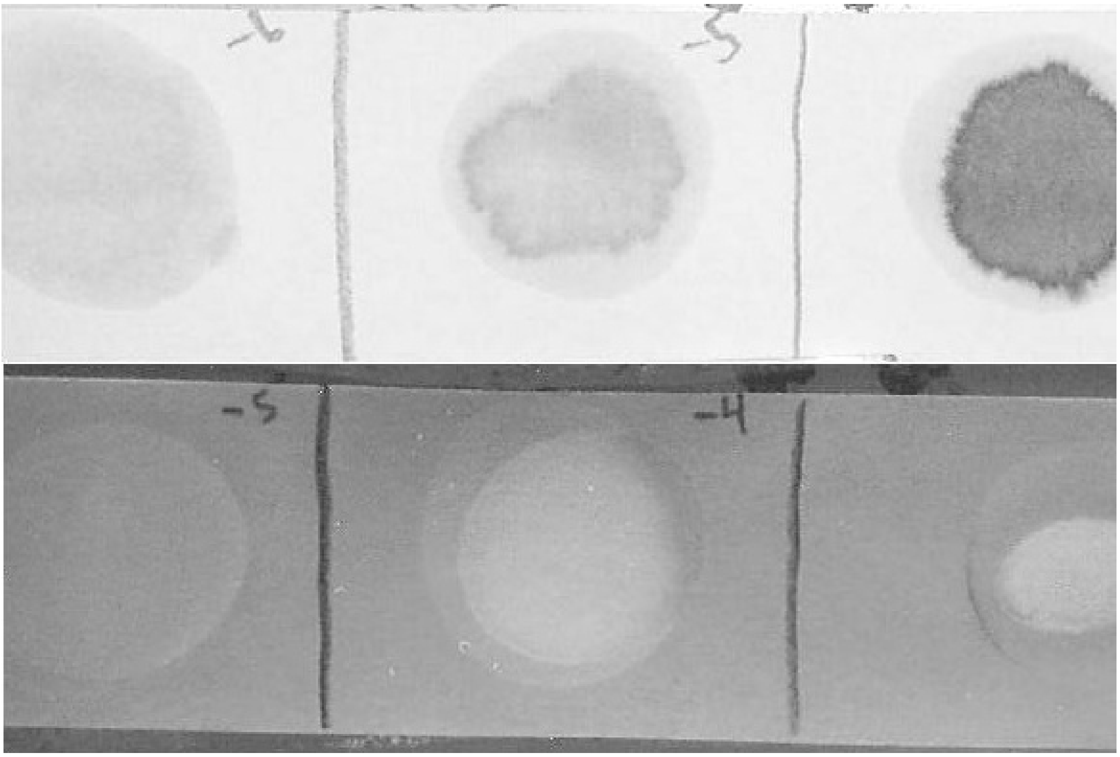

**FIGURE 5.** Colorimetric ETK$^{Plus}$ (top) and europium/OP photoluminescence (bottom) sensitivity comparison for smokeless powder. See text.

## Europium Luminescence Sensitization with Green Excitation

In practice, it would be of interest to be able to operate with the same green excitation of the ETK procedure when tagging explosives with europium complexes. In principle, this should be possible. In the sensitization, a critical feature is the spectral overlap between the fluorescence of the donor (OP or TTFA above) with the absorption of the acceptor (the Eu ion). The Eu ground state–emitting state energy gap (absorption) is at 579 nm. One would thus look for a donor (sensitizing ligand) that fluoresces at that wavelength and that can be excited by green light. Unfortunately, presently known sensitizing ligands are not suitable. In large measure, this is an issue of parity. The lanthanide f states and the p orbitals, which organic ligand states ultimately are made up of, have the same odd parity. The ligand-lanthanide energy transfer ultimately is determined by the odd parity electric dipole operator. Thus, the energy transfer is a forbidden process in the lanthanide/organic sensitizing ligand case[16]. It nonetheless occurs on UV excitation, because now higher lanthanide states are involved, which are less pure as f states than the lower ones and thus contain greater s and/or d even parity admixtures through which coupling to the ligand becomes possible. To explore a remedy to this visible excitation/sensitization problem, we examined the feasibility of utilizing highly emitting semiconductor nanoparticles (with emitting states of even parity) as sensitizers. These are also known as nanocomposites, nanocrystals, or quantum dots. Ideally, one would want CdSe nanoparticle sensitizers. They can be made to be excitable in the green and to emit at about 580 nm. Unfortunately, commercially available nanoparticles of this kind currently come biologically functionalized (e.g., Quantum Dot Corp., 26118 Research Rd., Hayward, CA 94545, www.Qdots.com) and are thus not useful for our purposes. However, we anticipate that suitably functionalized CdSe quantum dots will become available to us in the near future from the above-mentioned company. Since we have previously utilized and have on hand CdS nanoparticles for fingerprint detection[21], we utilized them here to examine the prospect of direct sensitization of the $^5D_0$ state of $Eu^{3+}$. Our CdS nanoparticles are in principle suitable for this sensitization in that they have a broad emission peaked at about 575 nm. That these nanoparticles require excitation in the UV (more efficiently in the deep than near UV) is not an obstacle to the present feasibility study in that the sensitization process is the primary issue for now, rather than the excitation. For the sensitization to occur, the sensitizer need not be chemically bound to the europium (this binding in the above-described lanthanide method mostly serves the purpose of excluding the luminescence-quenching water). It suffices that the donor and acceptor be in close proximity to each other. Accordingly, we first spotted nanoparticles on filter paper and then spotted the same place with europium to see whether energy transfer from the nanoparticle to europium takes place. Our CdS nanoparticles are functionalized with dioctyl sulfosuccinate (to prevent aggregation of the nanoparticles). Thus, they require a nonpolar solvent (we use heptane) for dissolution. The Eu-OP or Eu-TTFA complexes used (the OP or TTFA ligands are now primarily needed only to keep water molecules away from the Eu ion) are prepared in a polar solvent, however—methanol in our procedure. Thus, the nanoparticle and europium complex are, solvent-wise, not compatible. However, one can first prepare the Eu complex in methanol and then add heptane to the solution. Under vigorous stirring, the complex may migrate from the polar to the nonpolar solvent fraction via nonpolar functionalities of the ligand attached to the Eu ion. We found that this is not effective with OP as the ligand, but is with TTFA. The ligand now serves (in addition to water exclusion) the purpose of solubilization in nonpolar solvents. Thus, our study utilized Eu-TTFA, prepared in methanol, followed by addition of heptane, followed by vigorous stirring, followed by letting the mixture stand until the methanol and heptane fractions separate, and then decanting the heptane fraction that now contains the Eu complex. In our procedure, the CdS nanoparticles were smeared onto filter paper (when undissolved, they are in the form of a paste, rather than a powder) and then the heptane solution of the Eu complex was spotted at the nanoparticle site. For comparison purposes, the Eu complex was also spotted at a bare site. The two spots were then subjected to spectroscopic examination. The Eu-TTFA complex luminesces directly under UV excitation, with better efficiency under near UV than deep UV. The CdS nanoparticles luminesce directly as well under UV excitation, with better efficiency under deep UV than near UV. Thus, one would expect that the luminescence intensity ratio Eu Near/Eu Deep of the sharp 615-nm Eu emission peak would be the same at the two spot locales in absence of CdS-Eu sensitization, but different in presence of sensitization, larger at the bare spot locale (greater contribution to the Eu luminescence via CdS sensitization under deep UV than near UV). Our experiments utilized the earlier-mentioned hand-held UV lamp as excitation source and the earlier-mentioned 0.3-m monochromator/photomultiplier/photon counting apparatus for luminescence monitoring. Our ratio results were 6.5 (+– 0.3) at the bare spot locale and 4 (+_ 0.5) at the CdS/Eu locale, indicative indeed of CdS/Eu sensitization via the $^5D_0$ state.

## DISCUSSION

In the field detection scenario, the usual laboratory procedure of viewing luminescence in a darkened room (to exclude ambient light that masks the luminescence of interest) will not do. This is not a problem in that one simply places the trace explosive collecting swab, after chemical treatment (as in the usual field-testing procedure), in a (small) light-tight box with ports for the light source and for a filter-equipped eye piece for viewing. The device is easy to build, compact, and not expensive (excepting the light source). Our results with ETK indicate that the corresponding first-generation field device utilizing the battery-operated laser mentioned earlier is practically viable. We are in the process of constructing a prototype. One may imagine situations in which the field detection of trace explosive is to be done *in situ* (rather than by swabbing). In the photoluminescence context one must then contend with background color and/or fluorescence. Here, the lanthanide approach is of value in that time-resolved visualization allows the elimination of the background (when optical filtering is ineffective). Given the long luminescence lifetimes of lanthanides, this is not difficult to accomplish[9]. The light source is simply followed by a mechanical light chopper (relatively cheap, compact, and very easy to use). The chopper can easily be designed to provide the appropriate triggering to a proximity-focused microchannel plate image intensifier placed at the eye piece location of the above light-tight box and the time-resolved image is seen on the phosphor screen of the image intensifier. The light chopper blade would have two sets of openings, one to chop the exciting light and the other to provide the appropriate gate delay and gate width to the image intensifier via a light emitting diode (LED)–photosensor pair with associated electronics (standard fare for light choppers). In comparison with the above routine version of the device, one now has the added expense of the chopper and intensifier (not cheap), but it is unlikely that the time-resolved version would need to be deployed as widely as the routine version. Thus, cost would not be as much of an issue. As for excitation light sources, LEDs, diode lasers, and other compact sources are undergoing much development and can be expected in the future to impact field devices for trace explosives detection by photoluminescence techniques as well.

The advent of photoluminescent nanocrystals and nanocomposites offers additional prospects in the explosives arena. Here, one can foresee direct photoluminescence detection of explosive traces tagged with such nanoparticles, functionalized chemically with conjugating ligands to bind to the explosive. One would then have a nanoparticle method akin to the lanthanide method, in the above routine implementation, or in an appropriate time-resolved modality in which the laser/light chopper combination would be replaced by a pulsed laser, such as a frequency-tripled Nd: YVO4 laser operating at 355 nm. Such lasers have suitably short pulse widths (commensurate with $10^{-8}$-$10^{-6}$ s nanoparticle luminescence lifetimes), have high repetition rates (tens of kHz), provide average powers ranging from 100 mW to Watts, and operate on ordinary household current. The versions of the lower powers (100–500 mW) are not particularly bulky. They still are expensive, but perhaps not so much so as to be a show-stopper in the specialized time-resolved context. In terms of the above lanthanide strategy, these lasers act as if they were CW, given the long lanthanide luminescence lifetimes, and would be used directly, or together with the above-discussed simple mechanical light chopper for time-resolved purposes. The powers they provide are orders of magnitude larger than what one gets from the hand-held UV lamps discussed above, greatly aiding explosives detection sensitivity. Indeed, we have successfully used a laser of this kind (a prototype version meant for large-quantity industry use, Orion, manufactured by Iridex).

## ACKNOWLEDGMENT

This material is based upon work supported by the National Science Foundation and the Intelligence Technology Innovation Center through the joint “Approaches to Combat Terrorism” Program Solicitation NSF 03-569 (NSF 0345152).

## References

1. Almog, J., Kraus, S., and Glattstein, B. (1986) ETK — an operational explosive testing kit. *J. Energetic Mater.* **4**, 159–167.

2. Margalit, Y. (1996) Kit for detecting explosives. USA Patent 5,480,612, Jan. 2.
3. Kamyshny, A., Magdassi, S., Avissar, Y., and Almog, J. (2003) Water-soaked evidence: detectability of explosive traces after immersion in water. *J. Forensic Sci.* **48**, 312–317.
4. Jenkins, T.F. and Walsh, M.E. (1992) Development of field screening methods for TNT, 2,4-DNT and RDX in soil. *Talanta* **39**, 419–428.
5. Menzel, E.R. and Murdock, R.H. (2002) Explosives detection by photoluminescence techniques. Disclosure D-0390, Texas Tech University, Office of Technology Transfer and Intellectual Property, Lubbock, TX, May 29.
6. Menzel, E.R., Murdock, R.H., and Bouldin, K.K. (2003) Method for detecting trace explosives using photoluminescence. USA Prov. Patent Appl. No. 60/473,434, May 27.
7. Bouldin, K.K. Latent fingerprint and trace explosives detection by photoluminescence and time-resolved imaging. Ph.D. dissertation, May 2003, Texas Tech University.
8. Herod, D.W. and Menzel, E.R. (1982) Laser detection of latent fingerprints: ninhydrin followed by zinc chloride. *J. Forensic Sci.* **27**, 513–518.
9. Menzel, E.R. (1999) *Fingerprint Detection with Lasers*, 2nd ed., revised and expanded. Marcel Dekker, New York.
10. Dalrymple, B.E., Duff, J.M., and Menzel, E.R. (1977) Inherent fingerprint luminescence – detection by laser. *J. Forensic Sci.* **22**,106–115.
11. Yinon, J. and Zitrin, S. (1981) *The Analysis of Explosives*. Pergamon Press, Oxford.
12. Yinon, J. and Zitrin, S. (1993) *Modern Methods and Applications in Analysis of Explosives*. John Wiley & Sons, New York.
13. Saferstein, R. (1998) *Criminalistics, an Introduction to Forensic Science*, 6th ed. Prentice Hall, Upper Saddle River.
14. Strickler, S.J. and Berg, R.A. (1962) Relationship between absorption intensity and fluorescence lifetime of molecules. *J. Chem. Phys.* **37**, 814–821.
15. Horrocks, W.D., Jr. and Sudnick, D.R. (1981) Lanthanide ion luminescence probes of the structure of biological macromolecules. *Acc. Chem. Res.* **14**, 384–392.
16. Menzel, E.R. (1995) Laser Spectroscopy, Techniques and Applications. Marcel Dekker, New York.
17. Weissman, S.I. (1942) Intramolecular energy transfer, the fluorescence of complexes of europium. *J. Chem. Phys.* **10**, 214–217.
18. Forster, T. (1948) Zwischenmolekulare Energiewanderung und Fluoreszenz. *Ann. Phys.* **2**, 55–75.
19. Lock, E.R.A., Mazzella, W.D., and Margot, P. (1995) A new europium chelate as a fluorescent dye for cyanoacrylate pretreated fingerprints — EuTTAPhen: europium thenoyiltrifluoroacetone ortho-phenanthroline. *J. Forensic Sci.* **40**, 654–658.
20. Menzel, E.R. (2001) Fingerprint detection with photoluminescent nanoparticles. In *Advances in Fingerprint Technology*, 2nd ed., H. C. Lee and R. E. Gaensslen, Eds. CRC Press, Boca Raton.
21. Menzel, E.R., Savoy, S.M., Ulvick, S.J., Cheng, K.H. Murdock, R.H., and Sudduth, M.R. (2000) Photoluminescent semiconductor nanocrystals for fingerprint detection. *J. Forensic Sci.* **45**, 545–551.
